# Global nature run data with realistic high-resolution carbon weather for the year of the Paris Agreement

**DOI:** 10.1038/s41597-022-01228-2

**Published:** 2022-04-11

**Authors:** Anna Agustí-Panareda, Joe McNorton, Gianpaolo Balsamo, Bianca C. Baier, Nicolas Bousserez, Souhail Boussetta, Dominik Brunner, Frédéric Chevallier, Margarita Choulga, Michail Diamantakis, Richard Engelen, Johannes Flemming, Claire Granier, Marc Guevara, Hugo Denier van der Gon, Nellie Elguindi, Jean-Matthieu Haussaire, Martin Jung, Greet Janssens-Maenhout, Rigel Kivi, Sébastien Massart, Dario Papale, Mark Parrington, Miha Razinger, Colm Sweeney, Alex Vermeulen, Sophia Walther

**Affiliations:** 1 European Centre for Medium Range Weather Forecasts, Reading, RG2 9AX UK; 2 Cooperative Institute for Research in Environmental Sciences, University of Colorado Boulder, Boulder, CO USA; 3 NOAA, Global Monitoring Laboratory, Boulder, CO USA; 4 EMPA, Swiss Federal Laboratories for Materials Science and Technology, Überlandstrasse 129, Dübendorf, Switzerland; 5 Laboratoire des Sciences du Climat et de l’Environnement, CEA-CNRS-UVSQ, Université Paris Saclay, 91191 Gif-sur-Yvette CEDEX, France; 6 Laboratoire d’Aérologie, CNRS-Université de Toulouse, Toulouse, France; 7 Earth Sciences Department, Barcelona Supercomputing Center, Barcelona, Spain; 8 TNO, Department of Climate, Air and Sustainability, Utrecht, the Netherlands; 9 Max Planck Institute for Biogeochemistry (MPI-BGC), Jena, Germany; 10 European Commission, Joint Research Centre (JRC), Directorate for Energy, Transport and Climate, Air and Climate Unit, Via E. Fermi 2749, I-21027 Ispra, VA Italy; 11 Finnish Meteorological Institute, Sodankylä, Finland; 12 Dipartimento per la Innovazione nei Sistemi Biologici, Agroalimentari e Forestali, Università degli Studi della Tuscia, Largo dell’Università, Viterbo, Italy; 13 ICOS ERIC Carbon Portal, Sölvegatan 12, 22362 Lund, Sweden; 14 NOAA, Chemical Sciences Laboratory, Boulder, CO USA

**Keywords:** Atmospheric chemistry, Carbon cycle

## Abstract

The CO_2_ Human Emissions project has generated realistic high-resolution 9 km global simulations for atmospheric carbon tracers referred to as nature runs to foster carbon-cycle research applications with current and planned satellite missions, as well as the surge of *in situ* observations. Realistic atmospheric CO_2_, CH_4_ and CO fields can provide a reference for assessing the impact of proposed designs of new satellites and *in situ* networks and to study atmospheric variability of the tracers modulated by the weather. The simulations spanning 2015 are based on the Copernicus Atmosphere Monitoring Service forecasts at the European Centre for Medium Range Weather Forecasts, with improvements in various model components and input data such as anthropogenic emissions, in preparation of a CO_2_ Monitoring and Verification Support system. The relative contribution of different emissions and natural fluxes towards observed atmospheric variability is diagnosed by additional tagged tracers in the simulations. The evaluation of such high-resolution model simulations can be used to identify model deficiencies and guide further model improvements.

## Abstract (summary)

**Table:** 

Measurement(s)	atmospheric carbon dioxide, methane and carbon monoxide
Technology Type(s)	numerical simulation
Factor Type(s)	None
Sample Characteristic - Organism	long-lived greenhouse gases
Sample Characteristic - Environment	atmosphere
Sample Characteristic - Location	global atmosphere

## Background & Summary

Reducing human-made emissions of CO_2_ is at the heart of the climate change mitigation efforts in the Paris Agreement. In support of such efforts, the CO_2_ Human Emission (CHE) project (www.che-project.eu) has designed a prototype system to monitor CO_2_ fossil fuel emissions at the global scale. This challenging task requires the capability to detect and quantify the localised and relatively small signals of fossil fuel emissions in the atmosphere compared to the large variability of background CO_2_ concentrations not directly affected by local sources, and to distinguish anthropogenic sources from vegetation fluxes^1–3^. Using observations of atmospheric constituents to estimate emissions^4,5^ relies on a good understanding and accurate modelling of their atmospheric variability, which is largely determined by the weather-driven atmospheric transport together with surface biogenic fluxes and anthropogenic emissions. In the CHE project a library of nature runs of CO_2_ and species co-emitted with CO_2_ has been produced at different scales and with varying degrees of complexity^6^ which complements previous nature runs^7^.

Nature runs are very high-resolution simulations that mimic nature, in that they provide a realistic representation of processes of interest, in this case those modulating atmospheric CO_2_ variability. These simulations provide a reference for Observation System Simulation Experiments (OSSEs)^8^ Quantitative Network Design (QND)^9^. In OSSEs and QND studies, synthetic observations extracted from nature runs are used to assess the impact of different observing system configurations^10^. It is envisaged that such a monitoring system will rely on the use of a large variety of measurements including species co-emitted with CO_2_ that can help to isolate the fossil fuel emissions^3,11^. The future CO2M (Copernicus CO2 Monitoring) satellite mission is purposely designed to provide a high-resolution imaging capability to detect CO_2_ emission hotspots with high-precision observations of atmospheric CO_2_ concentrations^2,3,12^. CO2M will complement a constellation of satellites^4^ and a global *in situ* network^5^ to quantify the atmospheric CO_2_ variability from which emissions will be derived with atmospheric inversion systems.

**Fig. 1:**
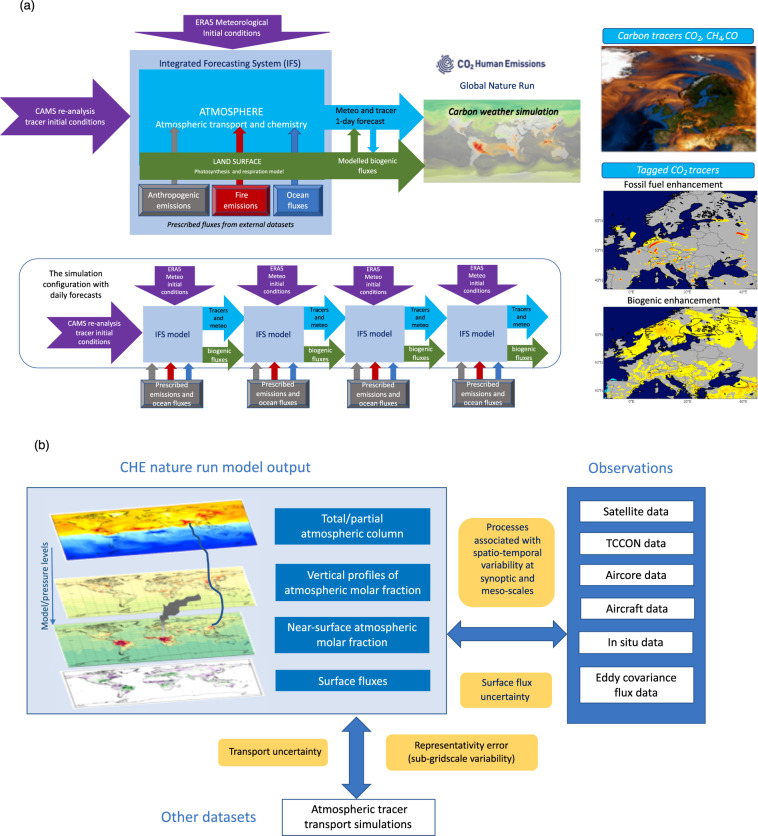
(**a**) Schematic of production framework for CHE nature run dataset (details of different components of the simulation in the text); (**b**) Overview of CHE nature run model output and strategy for comparison with different types of observations of carbon tracers and other relevant datasets such as lower resolution simulations. The differences between the CHE nature run and the various observations can be used to estimate and shed light into the different sources of uncertainty (orange boxes).

Simulating a realistic distribution of CO_2_ and co-emitters depends on the representation of the surface fluxes, chemical sources/sinks, and atmospheric transport. Here we use the Copernicus Atmosphere Monitoring Service (CAMS) high-resolution forecast of CO_2_, CH_4_ and CO (https://atmosphere.copernicus.eu/charts/cams/carbon-dioxide-forecasts) which has been demonstrated to produce realistic and accurate variability of carbon weather^13–15^. The configuration of the nature run is shown in Fig. 1. Note that the CHE nature run is a free-running tracer simulation unlike the CAMS high-solution forecast which is initialised daily from an atmospheric composition analysis.

The CHE nature run aims to support scientific studies that will shed light on the challenges of estimating CO_2_ emissions with the goal to build a CO_2_ monitoring and verification support capacity^3^. These challenges span a wide range of aspects from sparse observing systems, consistency between ocean/land observations from different satellite-view modes^16^, large variability in the biogenic signal^17^, large representativity errors in anthropogenic emissions^13^, transport errors^18^ and stringent requirements of high accuracy observations to estimate small signal with respect to large background values^16,19^. This global high-resolution dataset can provide a reference for testing different approaches to address those challenges.

## Methods

### Modeling framework

The CAMS high resolution forecasting system at the European Centre for Medium Range Weather Forecasts (ECMWF)^13,14,20^ has been used to produce the nature run dataset which includes simulations of CO_2_, CH_4_ and CO as illustrated in Fig. 1. It is based on the Integrated Forecasting System (IFS) model cycle 46R1 used to produce the operational weather forecast from June 2019 to June 2020^21^. The model has a reduced octahedral Gaussian grid^22^ with a resolution of Tco1279 (corresponding to approximately 9 km) and 137 model levels. The simulations have been produced by running a sequence of 1-day IFS forecasts of the carbon tracers and weather. The weather forecasts are initialized with state-of-the-art re-analysis of meteorological fields (ERA5)^23^. The atmospheric tracers start from the CAMS re-analysis^24,25^ initial conditions at the initial date of the dataset and from then onwards they are cycled from one forecast to the next in a free-running style. The different model components for the carbon weather forecast in the IFS, including the representation of the emissions for the different tracers, are listed in Table 1. All the emissions at the surface are prescribed except for the CO_2_ biogenic fluxes which are modelled online^26,27^, providing consistency between the response of fluxes to atmospheric conditions and tracer transport^28^. There are various differences with respect to the CAMS operational high-resolution forecast in 2015: improved anthropogenic emissions^29–31^ and natural CO_2_ ocean fluxes^32^; as well as an improved IFS model version^21^ and initial conditions^23–25^. The configuration of the simulations with daily re-initialisation of the weather forecast and free-running tracers ensures consistency of the tracer evolution throughout the simulation by avoiding jumps in their concentrations brought by the assimilation of observations in the analysis, while maintaining a realistic and accurate simulation of their atmospheric transport and variability of the underlying biogenic fluxes from the model^26,27^.

**Table 1:** Model components with emission datasets used as boundary conditions in the nature run simulation and prescribed atmospheric chemical sources/sinks.

Model components and emission datasets	Source	Horizontal and temporal resolution	References
*Tracer advection*	*IFS semi-Lagrangian scheme*	*Model resolution and time step*	^33,34,91,92^
*Tracer convective transport*	*IFS Tiedtke scheme*	*Model resolution and time step*	^93,94^
*tracer turbulent mixing*	*IFS boundary layer scheme*	*Model resolution and time step*	^95–97^
*CO*_*2*_ *biogenic fluxes*	*IFS CTESSEL A-gs with bias correction*	*Model resolution and time step*	^26,27^
*CO*_*2*_ *anthropogenic emissions*	*EDGARv*4*.5FT2015 annual emissions and EDGARv*4*.2FT20*1*0 monthly scaling factors CAMS-GLOB-TEMPO daily scaling factors for residential heating, CAMS-GLOB-AIR monthly emissions from aviation*	*0.1*^*o*^ × *0.1*^*o*^*, monthly, daily (residential sector)*	^29–31^
*CO*_*2*_ *ocean fluxes*	*Jena CarboScope v16*	*2.0*^*o*^ × *2.5*^*o*^*, daily fluxes averaged to monthly mean fluxes*	^32^
*CO*_*2*_*, CH*_4_*, CO fire emissions*	*GFAS v1.2*	*0.1*^*o*^ × *0.1*^*o*^*, daily*	^63^
*CH*_4_ *wetland fluxes*	*LPJ-HYMN climatology (*1*990-2008)*	*1*^*o*^ × *1*^*o*^*,monthly*	^98^
*CH*_4_ *anthropogenic emissions*	*CAMS-GLOB-ANT v2.1 (based on EDGAR4.3.2 in 2012 and EDGARv4.2FT2010 seasonal cycle for 2010)*.	*0.1*^*o*^ × *0.1*^*o*^*, monthly*	^99,100^
*CH*_*4*_ *other fluxes*	*Termites, wild animals, ocean fluxes and soil sink*	*1*^*o*^ × *1*^*o*^*,monthly*	^101–104^
*CO emissions*	*CAMS-GLOB-ANT v2.1*	*0.1*^*o*^ × *0.1*^*o*^*, monthly*	^99,100^
*CO chemistry*	*Linear CO chemistry scheme*	*Model resolution and time step*	^105^
*CH*_*4*_ *chemical loss rate*	*Climatological loss rate*	6^*o*^ × *4*^*o*^*, monthly*	^106^

Model resolution is around 9 km and model time step is 7.5 minutes.

### Model output

The standard parameters available from the CHE nature run dataset are listed in Table 2 and Table S1 in Supplementary Information file 1. Additional experimental tagged tracers are provided to characterize the atmospheric enhancement associated with the natural surface fluxes and anthropogenic emissions (Table 3). The enhancement can be computed by subtracting the concentrations of the background tracer without the specific emission/flux from the tracer concentration with the flux/emission. This assumes that the transport is linear. It is worth noting that artificial negative enhancements can occur in the vicinity of plumes due to numerical oscillations associated with the cubic interpolation of the advection scheme around very steep gradients. This can be considered a numerical error in the simulation. The CO_2_ tagged tracers are simulated without applying any mass fixer in order to ensure the signal comes only from the flux. The tagged tracers provide the enhancement during each 1-day simulation as they are re-initialised every day at 00UTC in order to avoid growing errors associated with the mass conservation^33,34^. This means the flux enhancement is reset to zero at 00 UTC. Detailed information on those tracers is provided in Table 4.

**Table 2:** Content of CHE nature run dataset with different parameter types and their associated data volume for the full year.

Parameters types and levels	Archived time step	Range of data volume
		per parameter in GBytes
Model levels parameters from level 1 (model top) to level 137 (model bottom)^*^	3-hourly	9–**7,373**
Pressure level parameters: 1, 2, 3, 5, 7, 10, 20, 30, 50, 70, 100 to 300 by 50, 400 to 700 by 100, 850, 925, 950, 1000 hPa	3-hourly	219–**1,241**
Parameters on surface layers	3-hourly	~146
1: 0–7 cm, 2: 7–21 cm, 3: 21–72 cm, 4: 72 cm–1.82 m		
2D surface fields	3-hourly	~55
2D prescribed daily emissions	daily	~7
2D prescribed monthly emissions	monthly	~0.24
3D prescribed monthly aviation emissions on model levels from level 1 (model top) to level 137 (model bottom)	monthly	~33

^*^Model levels can be converted to pressure levels p with the following equation p_i_ = p_sfc_B_i_ + A_i_ [in Pa] where p_sfc_ is surface pressure and A_i_ and B_i_ are static coefficients defined for each model level i (https://confluence.ecmwf.int/display/UDOC/L137 + model + level + definitions). The volume of atmospheric-tracer parameters has been highlighted in bold. The individual parameters are listed in Supplementary Information file 1 (Table S1).

**Table 3:** List of experimental CO_2_ tagged tracers from the CHE nature run dataset.

Tagged tracers	Parameter type	Parameter ID	Units	Enhancement processing (parameter IDs)
**CO**_**2**_ **tracer**	3D (model and pressure levels)	12.212	kg kg^-1^	
**CO**_**2**_ **tracer without fire emissions**	3D (model and pressure levels)	13.212	kg kg^−1^	3D Biomass burning (12.212-13.212)
**CO**_**2**_ **tracer without anthropogenic emissions**	3D (model and pressure levels)	14.212	kg kg^−1^	3D Anthropogenic (12.212-14.212)
**CO**_**2**_ **tracer without biogenic fluxes**	3D (model and pressure levels)	15.212	kg kg^−1^	3D Biogenic (12.212-15.212)
**CO**_**2**_ **tracer without ocean fluxes**	3D (model and pressure levels)	16.212	kg kg^−1^	3D Ocean (12.212-16.212)
**Total-column CO**_**2**_ **tracer**	2D (surface level)	112.212	kg m^−2^	
**Total-column CO**_**2**_ **tracer without fire emissions**	2D (surface level)	113.212	kg m^−2^	Column Biomass burning (112.212-113.212)^***^
**Total-column CO**_**2**_ **tracer without anthropogenic emissions**	2D (surface level)	114.212	kg m^−2^	Column Anthropogenic (112.212-114.212)^***^
**Total-column CO**_**2**_ **tracer without biogenic emissions**	2D (surface level)	115.212	kg m^−2^	Column Biogenic (112.212-115.212)^***^
**Total-column CO**_**2**_ **tracer without ocean fluxes**	2D (surface level)	116.212	kg m^−2^	Column Ocean (112.212-116.212)^***^

Each tracer is identified with a given experimental parameter ID. ^***^Note that the units of tagged tracers for the total column need to be converted from kg m^−2^ to ppm as described in 2D Atmospheric Composition parameters.

**Table 4:** Distribution of XCO_2_ anthropogenic enhancement (XCO_2__FF) accumulated over a 24-hour period from the CHE global nature run as mean number (and percentage in bold) of model cells with XCO_2__FF > 0.25 ppm (left columns) and XCO_2__FF > 0.50ppm (right columns).

	XCO_2__FF > 0.25 ppm Number model cells % model cells (Number clear-sky model cells) (% clear-sky model cells)			XCO_2__FF > 0.50 ppm Number model cells % model cells (Number clear-sky model cells) (% clear-sky model cells)		
	Land	Coast	Ocean	Land	Coast	Ocean
January	36,533 + /−2,458	41,018 + /−1924	15,689 + /−3,031	14,933 + /−1,312	18,178 + /−1,243	5,444 + /−1475
	**0.55 + /−0.04**	**0.62 + /−0.03**	**0.24 + /−0.05**	**0.23 + /−0.02**	**0.28 + /−0.02**	**0.08 + /−0.02**
	(11,194 + /−2,468)	(10,809 + /−3,101)	(3745 + /−1,466)	(4,995 + /−1,599)	(5,096 + /−1,915)	(1,471 + /−853)
	**(0.17 + /−0.04)**	**(0.16 + /−0.05)**	**(0.06 + /−0.02)**	**(0.08 + /−0.02)**	**(0.08 + /−0.03)**	**(0.02 + /−0.01)**
July	24,352 + /−1,235	28,203 + /−867	9,107 + /−2212	8,603 + /−583	11,325 + /−610	2,901 + /−954
	**0.37 + /−0.02**	**0.43 + /−0.01**	**0.14 + /−0.03**	**0.13 + /−0.01**	**0.17 + /−0.01**	**0.04 + /−0.01**
	(6,314 + /−1,288)	(5,746 + /−1,556)	(2,181 + /−1169)	(2,238 + /−613)	(2,289 + /−768)	(732 + /−554)
	**(0.10 + /−0.02)**	**(0.09 + /−0.02)**	**(0.03 + /−0.02)**	**(0.03 + /−0.01)**	**(0.03 + /−0.01)**	**(0.01 + /−0.01)**

The variability with respect to the mean number is shown by the +/− standard deviation. The statistics are also provided for clear-sky conditions, land, ocean and coastal regions, as these considerations are all relevant for satellite observations. Clear-sky model cells are defined with a cloud fraction threshold less than 10% over the 9 km × 9 km model cell; land cells have more than 99% land; ocean cells have less than 1% land and model cells over the coast have land between 1 and 99%.

Figure 1b provides an overview of the different types of model output from the CHE nature run dataset and how these can be compared to other datasets including various types of observations^5,35,36^ as well as atmospheric inversions/simulations of carbon tracers^9^. Such a comparison can shed some light on the different components of the uncertainty in the simulations of carbon tracers coming from the surface fluxes, the atmospheric transport and the representativity error associated with the limited model resolution^14^. A complementary lower resolution ensemble of simulations^18^ (25 km in the horizontal) has been also produced using the same model setup which provides information on emission uncertainty^30^, transport uncertainty and impact of meteorological uncertainty on biogenic fluxes. Two other major sources of uncertainty stem from the initial conditions of the carbon tracers at the beginning of the simulation^24,25^ and the biogenic flux model^26,27^. An estimation of these uncertainties is provided in the Technical Validation section.

### Example: Using tagged tracers to characterise anthropogenic plumes over land and ocean

In order to monitor anthropogenic CO_2_ emissions, it is crucial to observe the CO_2_ plumes emanating from the emission sources. These observations need to be based either targeted field campaign observations^13^ or on high resolution imaging satellites^10^. As satellites have different viewing geometries over land and ocean^16^, it is very important to understand how many of these plumes are located over land, ocean and coastal regions. Moreover, satellite observations only provide total column CO_2_ over cloud-free regions. Table 4 provides an example of statistics on the proportion of anthropogenic plumes accumulated over a 24-hour period over land/ocean and the proportion of plumes under cloudy conditions for January and July 2015. These fossil fuel tagged tracers and other tagged tracers associated with the biogenic fluxes, ocean fluxes and biomass burning emissions are all included in the CHE nature run dataset (see Table 3).

### Example: Insights into total column variability

**Fig. 2:**
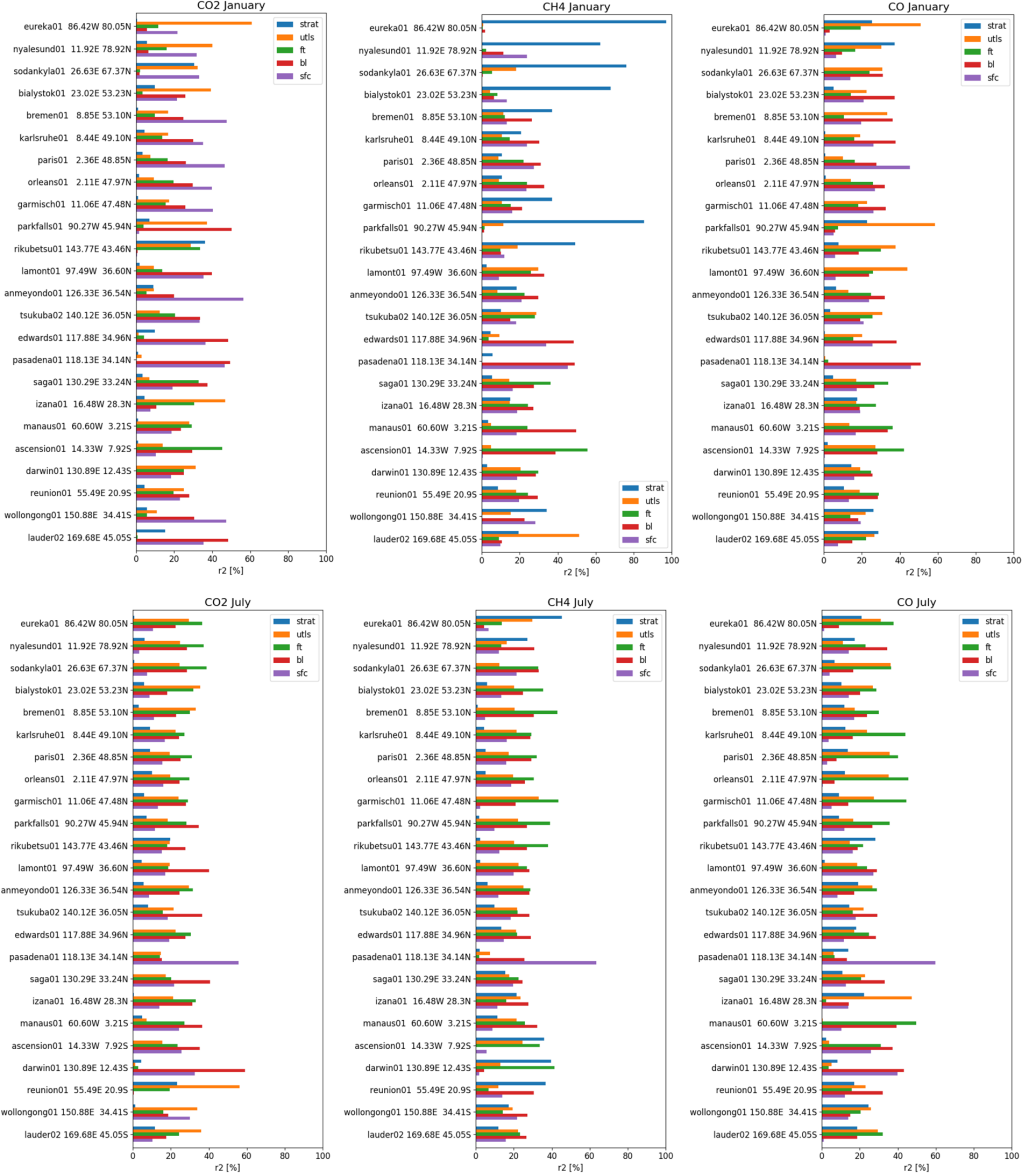
Coefficient of determination (r^2^) [%] of CO_2_, CH_4_ and CO total column with different partial layers in the atmospheric column in January and July 2015 at 24 TCCON sites (tccon.org). The atmospheric layers are defined as follows: from surface to 400 m (SFC), from 400 to 2 km (BL), from 2 km to 5 km (FT), from 5 km to 10 km (UTLS), from 10 km to the top of atmosphere (STRAT). All the column and partial column data have been detrended before calculating the coefficient of determination. All r^2^ values shown are statistically significant with p-value < 0.01 except when the r^2^ < 0.001.

The CO_2_, CH_4_ and CO observing system is based on *in situ* observations, at the surface or from tall towers, and remote sensing observations from ground-based stations or satellites providing partial/total column observations. There are currently very few vertical profile observations from aircrafts^37,38^ and Aircore measurements^36,39^ that can be used to link the two observation types. For low-resolution transport models assimilating both surface and total column observations in an atmospheric inversions framework, it can sometimes be challenging to combine the surface and total column variability for various reasons. These include errors in the remote sensing observations^16^, representation errors near the surface and model transport errors associated with vertical mixing^40^, atmospheric chemistry^41^, as well as long-range transport^42^ and the impact of stratospheric intrusions^43^. The global nature run can be useful to characterize the column variability of carbon tracers^44^ associated with transport. Figure 2 illustrates the potential use of the CHE nature run to explain the variability of XCO_2_, XCH_4_ and XCO at 24 TCCON sites (https://tccondata.org). The coefficient of determination shows that the variance of the total column can be explained by the different layers in the column in the nature run. When the column is well mixed, the contribution from the different layers is similar. At the sites where the influence of local emissions or natural fluxes is strong, the layers near the surface dominate the variability. Long-range transport in the free troposphere and upper troposphere/lower stratosphere also plays an important role, as depicted by the green/orange bars with higher r^2^ values than the near-surface layers in purple/red. The dataset can also be used to assess the important contribution of the stratosphere in the variability of XCH_4_^45^.

## Data Records

The CHE nature run dataset can be accessed through the ECMWF API following the examples provided in^46^. The data can be extracted on the native octahedral grid with the original resolution (tco1279, corresponding to approximately 9 km) or on a regular latitude/longitude grid at the required resolution of the user. Both grib and NetCDF formats are available. The dataset extends from 26 December 2014 to 31 December 2015. The list of contents is provided in Table 2. All meteorological and tracer fields and surface fluxes have been archived with 3-hourly time steps with respect to the 00 UTC initialization of the weather forecast. Step 0 of all the meteorological parameters represents the initial conditions taken from ERA5^23^. Atmospheric species (CO_2_, CH_4_ and CO) at step 0 are equivalent to tracers from the previous day at step 24, because they are free-running from one 1-day forecast to the next as illustrated in Fig. 1. Note that the emissions of CO and the CO_2_ emissions from aviation are not stored in the CHE nature run dataset, but they can be obtained from the Copernicus Atmosphere Data Store (https://ads.atmosphere.copernicus.eu).

## Technical Validation

The dataset is based on the state-of-the-art operational NWP and CAMS forecasting system^21,47^ which has been proven to produce reliable and accurate atmospheric CO_2_, CH_4_ and CO variability^13–15^. The CHE nature run focuses on 2015, a year characterised by a pronounced decrease in the terrestrial carbon sink associated with the strong El Niño Southern Oscillation (ENSO) of 2015-2016^48^ with droughts^49^, as well as fires in several regions, particularly over the tropics^50^. The larger than normal CO_2_ atmospheric growth rate in 2015^48,51^ and anomalously high fire emissions are well captured by the CHE nature run with a total global annual flux of 6.60 GtC (equivalent to 3.16 ppm/year from 1 January 2015 to 31 December 2015), which is close to the NOAA estimate of 2.99 + /−0.07 ppm/year (https://www.esrl.noaa.gov/gmd/ccgg/trends/global.html). The CO_2_ components of the budget include 9.29 GtC of anthropogenic emissions, 2.09 GtC of fire emissions, 2.10 GtC ocean sink and 2.69 GtC sink from land ecosystems. These values are consistent with the global carbon budget estimates^52^.

### Example: Evaluation of CO_2_ sources/sink by vegetation

**Fig. 3:**
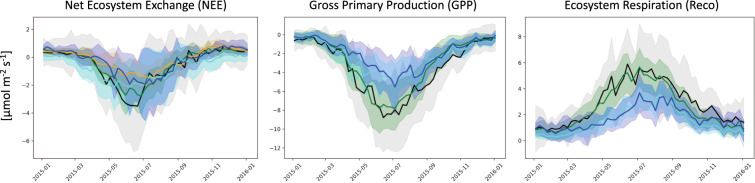
Mean seasonal cycle of CO_2_ biogenic fluxes [μmol m^−2^ s^−1^] at the 25 Eddy Covariance sites. FLUXNET2015^58^ observations [ICOS 2018 drought dataset^53^] are shown in black; the IFS modelled fluxes in cyan and the bias corrected fluxes used in the CHE nature run in blue; the CAMS inversion product^56,57,65^ (total flux –anthropogenic emissions) based on surface observations is shown in orange; and the CHE FLUXCOM product^54,55^ in green. The shading depicts the standard deviation across the 25 sites.

Biogenic CO_2_ fluxes associated with vegetation over land can dominate atmospheric CO_2_ variability on a wide range of time scales from diurnal, synoptic, seasonal to inter-annual^28^. They are a crucial component for the estimation of the background CO_2_ underlying the fossil fuel plumes from emission hotspots. This background CO_2_ has not been directly influenced by the plumes emanating from local anthropogenic sources, but it results from the larger-scale fluxes associated with biogenic sources and sinks over land. The European Eddy Covariance (EC) ecosystem flux data collected and processed by the Integrated Carbon Observation System (ICOS)^53^ are used to evaluate the uncertainty of modelled biogenic fluxes in the IFS (Fig. 3) which are bias-corrected^27^ in the CHE nature run. These modelled fluxes are also compared to other flux products, such as FLUXCOM^54,55^ (extended to include varying diurnal meteorology from ERA5) and the CAMS CO_2_ inversion (v18r3) product^56,57^. The EC data were processed and the Gross Primary Production (GPP) and ecosystem respiration (Reco) estimated using the standard methods applied in FLUXNET^58^ using the observed Net Ecosystem Exchange (NEE). Fig. 3 shows an overall underestimation of the seasonal cycle of NEE, GPP and Reco at the EC sites with typical errors of around 2 μmol m^−2^ s^−1^. Synoptic-scale errors are smaller while the diurnal cycle has larger errors of around 4 μmol m^−2^ s^−1^ (not shown in Fig. 3). This underestimation is exacerbated by the anomalously high NEE and Reco observed during the European drought in 2015 (Fig. SB7.3^49^). This type of evaluation can be used to understand the source of biogenic flux errors and improve the underlying biogenic models, as well as to quantify the uncertainty of prior fluxes for atmospheric inversions^59^.

### Example: Simulation and observation mismatch in the total column of CO_2_, CH_4_ and CO

**Fig. 4:**
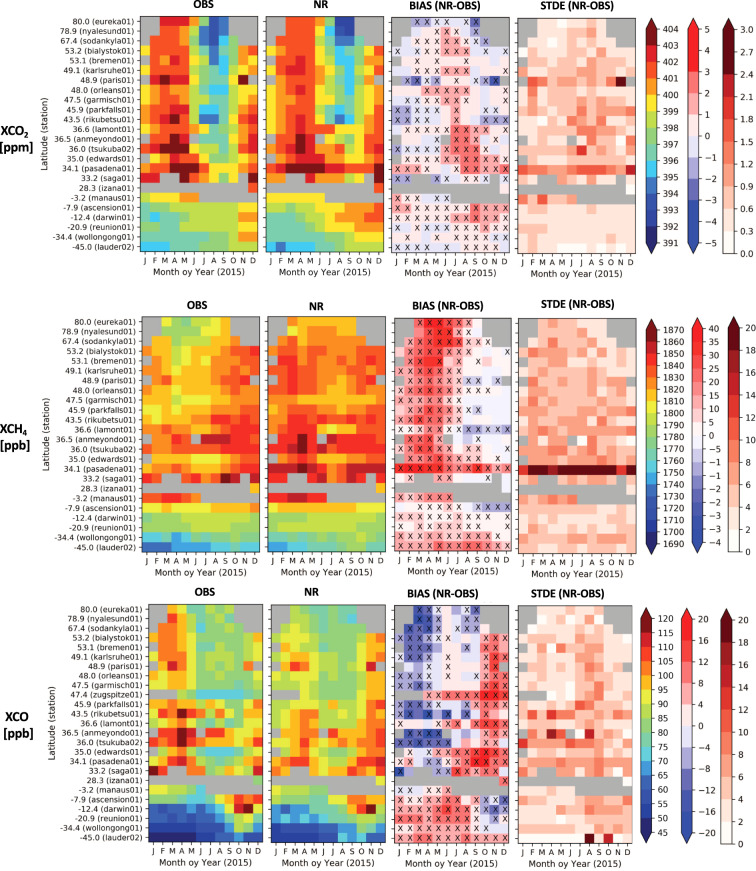
Evaluation of XCO_2_, XCH_4_ and XCO from the CHE nature run (NR). The nature run is compared to total column FTIR observation^35,60^ at the TCCON stations^67–90^ (OBS). The crosses indicate that the bias is statistically significant (p-value < 0.01).

The TCCON data^60^ which is widely used as a reference to evaluate biases in global measurement of CO_2_, CH_4_ and CO total column averages–referred to as XCO_2_, XCH_4_ and XCO–from space^16^ is used here to assess the inter-hemispheric gradient, seasonal cycle and synoptic day-to-day variability in the nature run dataset (Fig. 4). The large-scale patterns of variability on a monthly scale are generally well represented for the three species. The amplitude of the XCO_2_ seasonal cycle is underestimated at most TCCON sites, with the summer trough being 1 to 3 ppm higher than observed. This is consistent with the general underestimation of the biogenic sink during the growing season shown in Fig. 3. XCH_4_ is overestimated in spring/summer and underestimated in autumn/winter, due to errors in the seasonality of the chemical sink and emissions (e.g. wetlands, agriculture and biomass burning). XCO is underestimated in winter which is a common feature in many models and emission data sets^61^ and overestimated in summer/autumn, often caused by the biogenic emissions of isoprene, which have a large impact on southern hemisphere and global background values^62^ of CO. Other sources of error are associated with the chemical sources/sinks^61^ and fire emissions^63^, as 2015 was an extreme year for CO because of Indonesian fires in autumn^64^. Part of the bias shown in Fig. 4 also comes from the CO_2_, CH_4_ and CO initial conditions at the start of the nature run extracted from the CAMS re-analysis^24,25^. The random error in the sub-monthly variability (STDE in Fig. 4) - associated with surface fluxes/emissions and atmospheric transport - is generally below 1.5 ppm for XCO_2_, 10 ppb for XCH_4_ and 10 ppb for XCO, except at urban sites near emission hotspots such as Pasadena, Tsukuba and Paris.

### Example: Fine-scale structure in vertical profiles

**Fig. 5:**
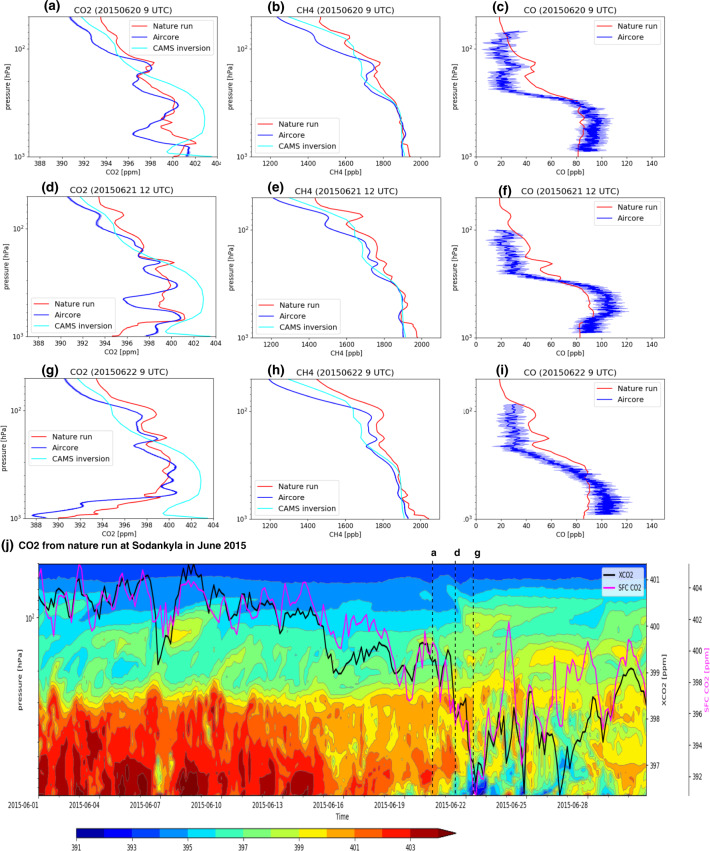
Examples of CO_2_ CH_4_ and CO vertical profiles from the CHE nature run at Sodankylä (67.37°N, 26.63°E). The nature run is compared to NOAA AirCore (v20201223) observations^36,39^ and the CAMS CO_2_ and CH_4_ inversion^57,65,66^ (**a**–**i**) during three days in June, depicted by the dashed lines in (**j**) where the nature run hovmöller plot for CO_2_ shows the temporal variability of the vertical profile at Sodankylä over the whole month of June. The solid black and magenta lines show the time series of XCO_2_ and near-surface CO_2_ averaged over the model levels from the surface to 400 m above the surface (SFC CO2) respectively.

The vertical profiles of CO_2_, CH_4_ and CO are illustrated in Fig. 5 with a comparison to AirCore observations^36,39^ from the National Oceanic and Atmospheric Administration (NOAA) Global Monitoring Laboratory and the lower-resolution CAMS surface *in situ* inversion dataset^57,65,66^. While most global transport models used in atmospheric inversion systems have too coarse horizontal and vertical resolution to be able to represent the fine-scale vertical structure, the CHE nature run is able to capture the small-scale anomalies along the atmospheric column from the surface up to the lower stratosphere (50 hPa). The profiles on three different consecutive days show the large variability associated with day-to-day synoptic transport, particularly for CO_2_. Capturing this type of vertical variability is important because it reflects the ability of atmospheric transport models to represent vertical mixing and long-range transport. Both need to be accurately represented in atmospheric inversions in order to accurately infer surface fluxes. Examples of anticorrelation between the near-surface CO_2_ and XCO_2_ are also shown in Fig. 5j (e.g. 7, 9, 15, 20, 21 and 24 June) which are associated with the advection of anomalously high/low CO_2_ air in the free troposphere (above 700 hPa) and the opposite decrease/increase of CO_2_ near the surface. This emphasizes the importance of tracer transport above the planetary boundary layer in explaining the variability of XCO_2_ also shown in Fig. 2.

## Supplementary information

Supplementary Information file 1

## Data Availability

The IFS forecast model and the Meteorological Archival and Retrieval System (MARS) software are not available for public use as the ECMWF Member States are the proprietary owners. However, the CHE global nature run dataset and the MARS data extraction features are freely available through ECMWF API (https://www.ecmwf.int/en/forecasts/access-forecasts/ecmwf-web-api) following a registration step (https://apps.ecmwf.int/registration/). The data can be accessed using python (https://www.python.org). The commands and steps required are detailed in the Supplementary Information file 1 (S2).
