# Supplementary material for: Global nature run data with realistic high-resolution carbon weather for the year of the Paris Agreement
Source: Sci Data. 2022 Apr 11;9:160. doi: 10.1038/s41597-022-01228-2 (PMC9001646; doi:10.1038/s41597-022-01228-2)
Supplement: Supplementary file 1 — Supplementary Information file 1 [file 41597_2022_1228_MOESM1_ESM.pdf]

Supplementary information for Global nature run data with realistic high-resolution carbon weather for the year of the Paris Agreement

By Agustí-Panareda et al.

Table of contents for supplementary materials

S1. List of parameters in CHE nature run dataset (Table S1, pages 2-3)
S2. Instructions Instructions for CHE nature run data access (pages 4-5)

Parameter	Parameter type	Parameter ID	Units
**2 metre dewpoint temperature**	single level (surface)	168.128	K
**2 metre temperature**	single level (surface)	167.128	K
**10 metre U wind component**	single level (surface)	165.128	$m\ s^{-1}$
**10 metre V wind component**	single level (surface)	166.128	$m\ s^{-1}$
**Accumulated carbon dioxide ecosystem respiration (REC)&**	single level (surface)	82.228	$kg\ m^{-2}$
**Accumulated carbon dioxide Gross Primary Production (GPP)&**	single level (surface)	81.228	$kg\ m^{-2}$
**Anthropogenic emissions of carbon dioxide&**	single level (surface)	69.210	$kg\ m^{-2}s^{-1}$
**Boundary layer height**	single level (surface)	159.128	m
**Carbon dioxide^**	model and pressure levels	61.210	$kg\ kg^{-1}$
**Carbon dioxide column-mean molar fraction^^**	single level (surface)	64.210	ppm
**Carbon monoxide^**	model and pressure levels	123.210	$kg\ kg^{-1}$
**Charnock**	single level (surface)	148.128	unitless
**Convective available potential energy**	single level (surface)	59.128	$J\ kg^{-1}$
**Convective inhibition**	single level (surface)	1.228	$J\ kg^{-1}$
**Convective precipitation**	single level (surface)	143.128	m
**Convective rain rate**	single level (surface)	218.228	$kg\ m^{-2}\ s^{-1}$
**Convective snowfall rate water equivalent**	single level (surface)	220.228	$kg\ m^{-2}\ s^{-1}$
**Direct solar radiation**	single level (surface)	47.128	$J\ m^{-2}$
**Divergence**	model and pressure levels	155.128	s^{-1}
**Downward UV radiation at the surface**	single level (surface)	57.128	$J\ m^{-2}$
**Evaporation**	single level (surface)	182.128	m of water equivalent
**Flux of carbon dioxide ecosystem respiration (instantaneous REC)&**	single level (surface)	85.228	$kg\ m^{-2}\ s^{-1}$
**Flux of carbon dioxide Gross Primary Production (instantaneous GPP)&**	single level (surface)	84.228	$kg\ m^{-2}\ s^{-1}$
**Flux of carbon dioxide Net Ecosystem Exchange (instantaneous NEE)&**	single level (surface)	83.228	$kg\ m^{-2}\ s^{-1}$
**Forecast albedo**	single level (surface)	243.128	(0-1)
**GPP coefficient from Biogenic Flux Adjustment System (bias correction scaling factor for GPP)**	single level (surface)	78.228	unitless
**Geopotential***	model and pressure levels	129.128	$m^2\ s^{-2}$
**High cloud cover**	single level (surface)	188.128	(0-1)
**Instantaneous 10 metre wind gust**	single level (surface)	29.228	$m\ s^{-1}$
**Instantaneous moisture flux**	single level (surface)	232.128	$kg\ m^{-2}\ s^{-1}$
**Instantaneous surface sensible heat flux**	single level (surface)	231.128	$W\ m^{-2}$
**Land-sea mask**	single level (surface)	172.128	(0-1)
**Large scale rain rate**	single level (surface)	219.228	$kg\ m^{-2}\ s^{-1}$
**Large scale snowfall rate water equivalent**	single level (surface)	221.228	$kg\ m^{-2}\ s^{-1}$
**Large scale precipitation**	single level (surface)	142.128	m
**Logarithm (natural) of surface pressure.**	model levels	152.128	**
**Low cloud cover**	single level (surface)	186.128	(0-1)
**Mean sea level pressure**	single level (surface)	151.128	Pa
**Medium cloud cover**	single level (surface)	187.128	(0-1)
**Methane^**	model and pressure levels	62.210	$kg\ kg^{-1}$
**Methane column-mean molar fraction^^**	single level (surface)	159.128	ppb
**Methane loss rate due to Radical hydroxyl (OH)**	model levels	71.210	s^{-1}
**Methane surface fluxes (excluding biomass burning) &**	single level (surface)	70.210	$kg\ m^{-2}\ s^{-1}$
**Ocean flux of carbon dioxide &**	single level (surface)	67.210	$kg\ m^{-2}\ s^{-1}$
**Photosynthetically active radiation at the surface**	single level (surface)	58.128	$J\ m^{-2}$
**REC coefficient from Biogenic Flux Adjustment System (bias correction scaling factor for REC)**	single level (surface)	79.228	unitless
**Relative humidity**	pressure levels	157.128	%
**Runoff**	single level (surface)	205.128	m
**Sea ice fraction**	single level (surface)	31.128	(0-1)
**Sea surface temperature**	single level (surface)	34.128	K
**Skin temperature**	single level (surface)	235.128	K
**Snow albedo**	single level (surface)	32.128	(0-1)
**Snow density**	single level (surface)	33.128	$kg\ m^{-3}$
**Snow depth**	single level (surface)	141.128	m of water equivalent
**Snow evaporation**	single level (surface)	44.128	m of water equivalent
**Snowfall**	single level (surface)	144.128	m of water equivalent
**Snowmelt**	single level (surface)	45.128	m of water equivalent

**Soil temperature level 1**	single level (surface)	139.128	K
**Soil temperature level 2**	single level (surface)	170.128	K
**Soil temperature level 3**	single level (surface)	183.128	K
**Soil temperature level 4**	single level (surface)	236.128	K
**Specific cloud ice water content**	model levels	246.128	$kg\ kg^{-1}$
**Specific humidity**	model and pressure levels	133.128	$kg\ kg^{-1}$
**Sunshine duration**	single level (surface)	189.128	s
**Surface latent heat flux**	single level (surface)	147.128	$J\ m^{-2}$
**Surface net solar radiation**	single level (surface)	176.128	$J\ m^{-2}$
**Surface net solar radiation, clear sky**	single level (surface)	210.128	$J\ m^{-2}$
**Surface net thermal radiation**	single level (surface)	177.128	$J\ m^{-2}$
**Surface net thermal radiation, clear sky**	single level (surface)	211.128	$J\ m^{-2}$
**Surface sensible heat flux**	single level (surface)	146.128	$J\ m^{-2}$
**Surface solar radiation downwards clear sky**	single level (surface)	129.228	$J\ m^{-2}$
**Surface solar radiation downwards**	single level (surface)	169.128	$J\ m^{-2}$
**Surface thermal radiation downwards clear sky**	single level (surface)	130.228	$J\ m^{-2}$
**Surface thermal radiation downwards**	single level (surface)	175.128	$J\ m^{-2}$
**Temperature**	model and pressure levels	130.128	K
**TOA incident solar radiation**	single level (surface)	212.128	$J\ m^{-2}$
**Temperature of snow layer**	single level (surface)	238.128	K
**Top net solar radiation**	single level (surface)	178.128	$J\ m^{-2}$
**Top net solar radiation, clear sky**	single level (surface)	208.128	$J\ m^{-2}$
**Top net thermal radiation**	single level (surface)	179.128	$J\ m^{-2}$
**Top net thermal radiation, clear sky**	single level (surface)	209.128	$J\ m^{-2}$
**Total cloud cover**	single level (surface)	164.128	(0-1)
**Total column carbon monoxide**	single level (surface)	127.210	$kg\ m^{-2}$
**Total column cloud ice water**	single level (surface)	79.128	$kg\ m^{-2}$
**Total column cloud liquid water**	single level (surface)	78.128	$kg\ m^{-2}$
**Total column ozone**	single level (surface)	206.128	$kg\ m^{-2}$
**Total column rain water**	single level (surface)	89.228	$kg\ m^{-2}$
**Total column snow water**	single level (surface)	90.228	$kg\ m^{-2}$
**Total column water**^^^	single level (surface)	136.128	$kg\ m^{-2}$
**Total column water vapour**	single level (surface)	137.128	$kg\ m^{-2}$
**Total precipitation**	single level (surface)	228.128	m
**Vertically integrated moisture divergence**	single level (surface)	213.128	$kg\ m^{-2}$
**Volumetric soil water layer 1**	single level (surface)	39.128	$m^3\ m^{-3}$
**Volumetric soil water layer 2**	single level (surface)	40.128	$m^3\ m^{-3}$
**Volumetric soil water layer 3**	single level (surface)	41.128	$m^3\ m^{-3}$
**Volumetric soil water layer 4**	single level (surface)	42.128	$m^3\ m^{-3}$
**Wildfire flux of carbon dioxide**&&	single level (surface)	80.210	$kg\ m^{-2}\ s^{-1}$
**Wildfire flux of methane**&&	single level (surface)	82.210	$kg\ m^{-2}\ s^{-1}$
**U-component of wind**	model and pressure levels	131.128	$m\ s^{-1}$
**V-component of wind**	model and pressure levels	132.128	$m\ s^{-1}$
**Vertical velocity**	model and pressure levels	135.128	$Pa\ s^{-1}$
**Vorticity (relative)**	model and pressure levels	138.128	s^{-1}

Table S1. List of parameters from the IFS CHE nature run dataset excluding the carbon monoxide surface emissions [$kg\ m^{-2}\ s^{-1}$] and carbon dioxide aviation 3D fluxes [$kg\ m^{-2}\ s^{-1}$] which are available from https://ads.atmosphere.copernicus.eu/cdsapp#!/dataset/cams-global-emission-inventories. Each field is identified with a given parameter. Note that the units of tracers for the total column need to be converted from kg m-2 to ppm as described in 2D Atmospheric Composition parameters. Details of model levels, pressure levels and surface layers are provided in Table 2. ^ To conversion of $kg\ kg^{-1}$ to dry molar fraction the mixing ratios should be multiplied by M_{air}/M_{tracer} where M_{air}=28.9644 is the molar mass of dry air, M_{tracer} is the molar mass of the tracer (44.0095 g/mol for carbon dioxide 16.04 for methane, 28.01 g/mol for carbon monoxide. ^^ Carbon dioxide and methane column-mean molar fractions should be treated as dry molar fractions. ^^^Unit conversion of CO total column in kg m-2 to averaged-column dry molar fraction [ppb] require the factor [10^9 x g x M_{air}]/ [M_{co} x exp(lnsp)], where g=9.80665 (the earth gravity acceleration), M_{air}=28.9644 is the molar mass of dry air, M_{co}=28.01 is the molar mass of carbon monoxide, and lnsp is the logarithm of surface pressure. *The geopotential height can be calculated by dividing the geopotential by the Earth's gravitational acceleration, g; orography can be extracted from geopotential height at model level 1.** Surface pressure [Pa] can be obtained by computing the exponential of the logarithm of surface pressure. &Note that the sign of the surface fluxes and emissions towards atmosphere are negative and sinks towards surface are positive. &&Wildfire emissions are an exception with positive sign.

S2. Instructions for CHE nature run data access

The CHE global nature run dataset and the MARS data extraction features are freely available through ECMWF API (https://www.ecmwf.int/en/forecasts/access-forecasts/ecmwf-web-api) following a registration step (https://apps.ecmwf.int/registration/). The data can be accessed using python (https://www.python.org). The commands and steps required are detailed below.

- **Login** to https://www.ecmwf.int/en/forecasts/access-forecasts/ecmwf-web-api (top right corner)
- **Install ECMWF key.**

  The key information can be obtained from https://api.ecmwf.int/v1/key/

  1. Save your API key credentials into a file

  Copy / paste the text below into a blank text file and save it to your $HOME directory as **.ecmwfapirc** (If you use Windows, you have to put the file in C:\Users\<USERNAME>\.ecmwfapirc)

```
{
  "url": "https://api.ecmwf.int/v1",
  "email": "<EMAILADDRESSFROMREGISTRATION>",
  "key": "<KEY>"
}
```

  2. Set these environment variables before executing the Python script:

```
export ECMWF_API_URL="https://api.ecmwf.int/v1"
export ECMWF_API_KEY="21a9e24ff8315300210da27365fa1c3c"
export ECMWF_API_EMAIL="anna.agusti-panareda@ecmwf.int"
```

- **Install client libraries**
  The client currently supports both Python 2.7.x and Python 3. The ecmwf-api-client library was added to the Python Package Index (PYPI): https://pypi.org/project/ecmwf-api-client/

```
pip install --user ecmwf-api-client
```

- **Use python script to extract dataset**. Examples of python scripts for model levels, pressure levels and surface data are shown below.

```
from ecmwfapi import ECMWFDataServer
server = ECMWFDataServer(url="https://api.ecmwf.int/v1",key="<KEY>",email="<EMAILADDRESS>")

server = ECMWFDataServer()

# Extract model level data
# Carbon dioxide and methane mass mixing ratios on the lowest (level 1 is the top of
# the atmosphere) model level for all the forecast steps for 1 July 2015 interpolated
# to 0.1 x 0.1 deg regular latitude-longitude grid

server.retrieve({
    "class": "rd",
    "dataset": "research",
    "date": "2015-07-01",
    "expver": "ha58",
    "grid": "0.1/0.1",
    "levelist": "1/to/137",
    "levtype": "ml",
    "param": "210061/210062",
```

```
    "step": "0/to/24/by/3",
    "stream": "oper",
    "target": "ml_output.nc",
    "format": "nc",
    "time": "00:00:00",
    "type": "fc",
})
# Extract pressure level data
# Carbon dioxide and methane mass mixing ratios on all the pressure levels for all the
# forecast steps for 1 July 2015 interpolated to 0.1 x 0.1 deg regular latitude-longitude grid

server.retrieve({
    "class": "rd",
    "dataset": "research",
    "date": "2015-07-01",
    "expver": "ha58",
    "grid": "0.1/0.1",
    "levelist": "1/2/3/5/7/10/20/30/50/70/100/150/200/250/300/400/500/600/700/850/925/950/1000",
    "levtype": "pl",
    "param": "210061/210062",
    "step": "0/to/24/by/3",
    "stream": "oper",
    "target": "pl_output.nc",
    "format": "nc",
    "time": "00:00:00",
    "type": "fc",
})
# Extract column data
# Carbon dioxide and methane column mean molar fractions for all the forecast steps for 1 January
# 2015 interpolated to 0.1 x 0.1 deg regular latitude-longitude grid

server.retrieve({
    "class": "rd",
    "dataset": "research",
    "date": "2015-01-01",
    "expver": "ha58",
    "grid": "0.1/0.1",
    "levtype": "sfc",
    "param": "64.210/65.210",
    "step": "0/to/24/by/3",
    "stream": "oper",
    "target": "output.grib",
    "time": "00:00:00",
    "type": "fc",
})

# Extract surface data
# Natural biosphere flux and anthropogenic emissions of carbon dioxide for all the forecast steps
# for 31 December 2015 interpolated to 0.1 x 0.1 deg regular latitude-longitude grid
server.retrieve({
    "class": "rd",
    "dataset": "research",
    "date": "2015-12-31",
    "expver": "ha58",
    "grid": "0.1/0.1",
    "levtype": "sfc",
    "param": "68.210/69.210",
    "step": "0/to/24/by/3",
    "stream": "oper",
    "target": "output.grib",
    "time": "00:00:00",
    "type": "fc",
})
```
